# Issues with radiofrequency heating in MRI

**DOI:** 10.1120/jacmp.v15i5.5064

**Published:** 2014-09-08

**Authors:** Jihong Wang

**Affiliations:** ^1^ Department of Radiation Physics and Department of Imaging Physics The University of Texas MD Anderson Cancer Center Houston TX USA

To the Editor:

In recent years, the use of magnetic resonance imaging (MRI) has been increasing in patients with implanted devices, such as cardiac pacemakers and neurostimulators, which used to preclude the use of MRI owing to patient safety and device malfunction concerns.^1^, ^2^, ^3^, ^4^, ^5^, ^6^ In the past few years, however, more of these implant devices have become MR‐compatible and can function properly during MRI studies. For these devices, one of the remaining limiting factors and safety considerations is radiofrequency (RF) heating, particularly in the conductive part of the implanted device which may damage the surrounding tissues. To allow the use of MRI with these types of devices, manufacturers typically specify the conditions under which imaging sequences can be safely used, including the maximum specific absorption rate (SAR) values and gradient strengths. For SAR values, clinical users often rely solely on the SAR values reported by the MRI system for specific MRI sequences. Therefore, the accuracy and consistency of the SAR values reported by the MRI system are become more relevant and critical for patient safety. However, to the best of our knowledge, these SAR values are not routinely verified or validated independently by clinical users anywhere in the today's clinical practice.

Although the physical principles of RF heating are simple and straightforward, accurate calculation of the SAR (measured in W/kg) is complicated by many factors, including patient size, heterogeneity of tissue conductivity, and differences in the RF power distribution profiles of the various MRI scanning sequences, as well as the specific scanning parameters. In general, SAR values increase with patient body weight. However, for the most part, the calculation of SAR values is proprietary for each MRI system manufacturer. Consequently, no independent validation or verification is available to clinical users, who must completely rely on the SAR value reported by the vendor.

**Figure 1:**
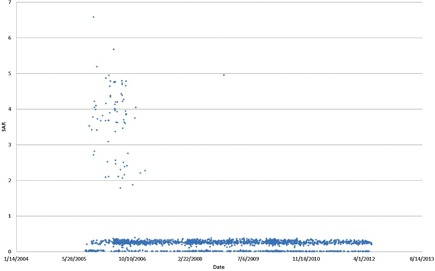
Specific absorption rate (SAR) values of three‐plane localizer scans.

In a recent survey of the SAR values for clinical MRI spine studies at our institution, we found some inconsistencies and possible inaccuracies in the reported SAR values. As shown in Fig. 1, for the reported SAR values for the three‐plane localizer scans at 1.5 T, some of the SAR values for the localizer sequences were quite high (even beyond the US Food and Drug Administration safety limits). This was not expected, and was most likely due to a calculation error or a bug in the SAR value calculation and reporting program, rather than actual high SAR values in the scans.

**Figure 2:**
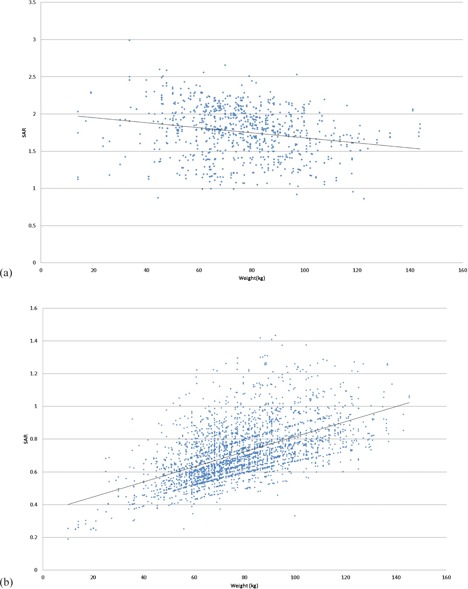
Specific absorption rate (SAR) values for T2 sagittal scans on 3‐T (a) and 1.5‐T systems (b). Note the negative correlation between SAR and patient body weight for scans done on the 3‐T systems compared with the positive correlation for scans done on the 1.5‐T systems.

Additionally, we noticed some inconsistencies in the relationship between reported SAR values and patient body weight for the 3‐T systems from one MRI vendor. As shown in Fig. 2(a), the SAR values were negatively correlated with patient body weight, whereas in theory they should be positively correlated with body weight (as shown in Fig. 2(b) for the 1.5‐T scanners from the same vendor).

Our survey results cast doubt on the accuracy and reliability of reported SAR values, and indicate that SAR reporting should be standardized to ensure consistency and reliability in reported SAR values, which are becoming more important for clinical decisions involving the use of MRI in patients with implanted devices. We hope to raise awareness of this issue among clinical users who currently completely rely on these reported SAR values. We also hope that this will be the first step to bring the medical physics community together to demand openness and standardization from MRI manufacturers in the calculation of SAR values, and that this will ultimately lead to some kind of independent validation of SAR values, as is currently done for radiation doses in CT studies.
